# Impact of Cryopreservation on Viability, Phenotype, and Functionality of Porcine PBMC

**DOI:** 10.3389/fimmu.2021.765667

**Published:** 2021-11-29

**Authors:** Yanli Li, Enric Mateu, Ivan Díaz

**Affiliations:** ^1^ Departament de Sanitat i Anatomia Animals, Universitat Autònoma de Barcelona (UAB), Bellaterra, Spain; ^2^ Centre de Recerca en Sanitat Animal, Institut de Recerca en Tecnologies Agroalimentàries (IRTA-CReSA), Bellaterra, Spain; ^3^ World Organisation for Animal Health (OIE) Collaborating Centre for the Research and Control of Emerging and Re-Emerging Swine Diseases in Europe (IRTA-CReSA), Bellaterra, Spain

**Keywords:** PBMC, Elispot, proliferation, porcine, cryopreservation, antigen-specific response

## Abstract

The use of frozen peripheral blood mononuclear cells (PBMC) is common in immunological studies. The impact of freezing PBMC has been assessed using human and mice cells, but little information is available regarding domestic animals. In the present study, the phenotype and functionality of frozen porcine PBMC were examined. In a preliminary experiment, three freezing media: fetal bovine serum plus 10% dimethyl sulfoxide, PSC cryopreservation kit, and Cryostor CS10, were compared regarding the preservation of cell viability and the response of PBMC to mitogens after thawing. After being stored one month in liquid nitrogen, cell viability was above 89% for all freezing media. The ELISPOT IFN-gamma (IFN-γ) results in response to PHA and of IgG ELISPOT in response to R848+IL-2 were similar to those obtained using fresh PBMC. In the second set of experiments, PBMC were obtained from five pigs vaccinated against *Porcine reproductive and respiratory syndrome virus* (PRRSV) and then frozen using Cryostor CS10. Recovered cells were phenotyped by flow cytometry using anti-CD3, CD4, CD8, and CD21 antibodies and were used to assess the PRRSV-specific responses in a proliferation experiment, an IFN-γ ELISPOT, and an IgG ELISPOT, and compared to the results obtained with fresh cells. The antigen-specific responses of frozen cells were significantly (p<0.05) impaired in the proliferation assay, particularly for CD4/CD8 double-positive T-cells and for CD21+ cells. Freezing resulted in decreased proliferation when Con A, but not PHA, was used. In ELISPOT, cryopreservation resulted in a decreased frequency of IFN-γ-secreting cells in response to PRRSV (p<0.05) but the response to PHA was not affected. No differences were observed in the IgG ELISPOT after polyclonal activation. Taken together, cryopreservation of porcine PBMC had a significant impact on the magnitude of recall antigen responses and therefore, it may affect the response of effector/memory cells but seems not to have a major impact on naïve T-cells. These results may help to the better use of frozen porcine PBMC, and to the interpretation of the results obtained from them.

## Introduction

Measuring B- and T-cell responses against particular antigens is pivotal to understand how the adaptive immune response develops in the course of an infection, or after vaccination. However, examination of B and T-cell responses in experimental studies is difficult for several reasons. Under experimental conditions, several groups or batches of animals must be examined considering individual variations. In the case of large animals, implies housing and management in special facilities that have limited allocation capacity. Under farm conditions, although more animals can be examined, the preservation and processing of samples become the main challenge, as farms are usually far from research institutes. Also, it is not always possible to obtain samples, such as lymphocytes from lymph nodes, that can only be collected after killing the animal, due to ethical and economic reasons. Thus, separation of peripheral blood mononuclear cells (PBMC) after bleeding an animal is a common strategy that allows sampling numerous animals at the same time. This is convenient when performing a longitudinal follow-up study (e.g., to evaluate the variation of the adaptive response over time) and elucidating B- and T-cell responses by means of cryopreservation of PBMC. Besides, frozen replicas of PBMC from a given animal can be used to retest or to perform additional tests if necessary.

Cryopreserved PBMC are extensively used in human immunology. However, PBMC, as other cell types, are sensitive to the freezing process. It is well-known that cells can be damaged during freezing, mainly by the intracellular formation of ice crystals -which can mechanically damage the cell-, or by the osmotic imbalance between the intra- and extra-cellular space, resulting in dehydration and shrinkage. In addition, channels formed by the residual unfrozen medium outside the cells could also damage them (1). The impact of cryopreservation on T and B-cell subsets of PBMC continues to be a controversial issue. For human PBMC, some reports have not observed substantial changes between fresh and frozen cells in terms of phenotype proportions and functionality, such as the antigen-specific responses or the response to mitogens (2–6). Nevertheless, others claimed that cryopreservation alters the proportions of human PBMC subsets (7, 8), the antigen-specific IFN-γ responses using whole PBMC or specific T-cell subsets (9, 10), and impairs the proliferation after mitogen or antigen stimulation as well (3).

In the case of pigs, frozen PBMC are frequently used as well; however, few articles have analyzed the effects of cryopreservation (11, 12). Koch et al. (11) indicated that cryopreservation could impair some immunological functions, such as proliferation after PHA-stimulation, while Li et al. (12) found that impact on PBMC stimulated by PMA was disparate, from a significantly lower production of IL-6 in frozen cells to a significantly higher production of IFN-γ in frozen PBMC compared to fresh PBMC.

The present study aimed at evaluating the impact of cryopreservation on porcine PBMC in terms of viability, T- and B-cell subset proportions and functionality after mitogen- or antigen-specific stimulation. In a preliminary study, three cryopreservation media were compared to assess the best method to allow the highest survival rate.

## Materials and Equipment

### Kits

Porcine IgG ELISPOT^BASIC^ kit (HRP) including capture antibody MY91/145, biotinylated antibody MT78/145, streptavidin-peroxidase and TMB substrate, 3151-2H, Mabtech, Nacka Strand, Sweden CellTrace™ Violet Cell Proliferation Kit, C34557, Thermo Fisher Scientific, Waltham, Massachusetts, US

### Cell Media

RPMI Medium 1640 with HEPES wo L-Gln (RPMI-1640), H3BE04-558F, Lonza, Basilea, Switzerland

L-Glutamine 200 mM, 25030024, Thermo Fisher Scientific, Waltham, Massachusetts, US

Non-essential amino acids solution (100x), 11140035, Thermo Fisher Scientific, Waltham, Massachusetts, US

Sodium Pyruvate (100 mM), S-8636, Millipore Sigma, Saint Louis, Missouri

2-Mercaptoethanol, M6250-10ML, Merck, Darmstadt, Germany

Penicillin-Streptomycin (10,000 U/mL), 15140122, Thermo Fisher Scientific, Waltham, Massachusetts, US

Gentamicin (50 mg/mL), 15750045, Thermo Fisher Scientific, Waltham, Massachusetts, US

Fetal Bovine Serum (FBS), 35-079-CV, Corning, New York, US

**Supplemented medium-1 (SM1):** RPMI 1640 plus: 1mM L-Glutamine, 1 mM non-essential amino acids, 1 mM sodium pyruvate, 5 mM 2-mercaptoethanol, 50,000 IU/l penicillin 1, 50 mg/l streptomycin, 50 mg/l gentamicin and 10% FBS

**Supplemented medium-2 (SM2):** RPMI 1640 containing 10% FBS

### Buffers and Others

Cytiva HyClone™ Phosphate Buffered Saline (PBS), 10462372, Thermo Fisher Scientific, Waltham, Massachusetts, US

Dulbecco’s phosphate-buffered saline (DPBS), 14040133, Thermo Fisher Scientific, Waltham, Massachusetts, US

Horse Serum, New Zealand origin, 16050122, Thermo Fisher Scientific, Waltham, Massachusetts, US

Sodium bicarbonate (NaHCO_3_), 1.06329, Merck, Darmstadt, Germany

Sodium carbonate monohydrate (Na_2_Co_3_), 230952-100gr, Merck, Darmstadt, Germany

Bovine Serum Albumin (BSA), A7906-100G, Merck, Darmstadt, Germany

Tween^®^ 20, P1379, Merck, Darmstadt, Germany

Supplemented DPBS (blocking solution for flow cytometry): DPBS containing 5% FBS and 5% horse serum

**Carbonate–bicarbonate buffer (sterile):** 4.3 g NaHCO_3_ and 5.3 g Na_2_Co_3_ in 1 L distilled water (pH 9.4)

**Standard diluent buffer:** 5 g BSA and 1 mL Tween-20 in 1L PBS (pH 7.4)

**Wash buffer for ELISPOT:** 1 ml Tween 20 in 1 L distilled water (pH 7.4)

**Wash buffer for flow cytometry:** DPBS containing 2% FBS

Histopaque 1.077, 10771-500ML, Merck, Darmstadt, Germany

Trypan blue, T6146, Merck, Darmstadt, Germany

### Cryopreservation Products

Homemade freezing medium: 90% FBS and 10% Dimethyl sulfoxide Hybri-Max™ (DMSO), D2650-100ML, Merck, Darmstadt, Germany

PSC Cryopreservation kit, A2644601, Thermo Fisher Scientific, Waltham, Massachusetts, US

CryoStor^®^ CS10, 07955, Stemcell Technologies, Vancouver, Canada

### Mitogens and Others

Concanavalin A (ConA) from *Canavalia ensiformis*, C2010, Merck, Darmstadt, Germany

Lectin from *Phaseolus vulgaris* (red kidney bean) (PHA), L1668-5MG, Merck, Darmstadt, Germany

R848 included in the Porcine IgG ELISPOT^BASIC^ kit, 3151-2H, Mabtech, Nacka Strand, Sweden

Recombinant Porcine IL-2 Protein, 652-P2-020/CF, R&D Systems, Minneapolis, Minnesota, USA

### Antibodies, Streptavidin and Substrate

#### Antibodies for IFN-γ ELISPOT

Purified mouse Anti-Pig IFN-γ porcine, clone P2G10, 559961, final dilution 1:100, BD Biosciences Pharmingen, San Jose, California, US

Biotin Mouse Anti-Pig IFN-γ, clone P2C11, 559958, final dilution 1:1000, BD Biosciences Pharmingen, San Jose, California, US

#### Antibodies for Flow Cytometry

Mouse anti pig CD3, clone PPT3, MCA5951GA, final dilution 1:200, Bio-Rad, Hercules, California, US

PE Rat Anti-Mouse IgG1, clone A85-1, 562027, final dilution 1:800, BD Biosciences Pharmingen, San Jose, US

FITC mouse anti-pig CD4α, clone 74-12-4, 559585, final dilution 1:100, BD Biosciences Pharmingen, San Jose, US

Alexa Fluor^®^ 647 Mouse Anti-Pig CD8α, clone 76-2-11, 561475, final dilution 1:100, BD Biosciences Pharmingen, San Jose, US

Mouse Anti-Porcine CD21-PE, clone BB6-11C9.6, 4530-09, final dilution 1:100, SouthernBiotech, Birmingham, Alabama, USA

#### Streptavidin and Substrate

Streptavidin HRP (ELISA GD), SNN2004, Thermo Fisher Scientific, Waltham, Massachusetts, US

TMB substrate for ELISpot, 3651-10, Mabtech, Nacka Strand, Sweden

### Field Virus and Vaccine

*Porcine reproductive and respiratory syndrome virus* (PRRSV) strain 3267 (batch 40) belonged to our PRRSV bank (13), UAB, Barcelona, Spain. It was produced and titred in porcine alveolar macrophages obtained from three-weeks old PRRSV-negative piglets.

PORCILIS^®^ PRRS, modified live-virus PRRSV vaccine, MSD Animal Health, Madison, New Jersey, Us

### Others

SepMate™ 50 (IVD), 85450, Stemcell Technologies, Saint Égrève, France

Frosty™ Freezing Container, 5100-0001, Thermo Fisher Scientific, Waltham, Massachusetts, US

96-Well, Cell Culture-Treated, U-Shaped-Bottom Microplate, 3799, Corning, New York, US

MultiScreen-HA filter plate MAHAS4510, MAHAS4510, Merck, Darmstadt, Germany

### Software and Equipment

FCS Express 7, *de novo* Software, Glendale, California, US

Statsdirect Statistical software 3.3.5, Statsdirect LTD, Birkenhead, United Kigndom

GraphPad Prism 9.1.2, GraphPad Software Inc., San Diego, California, US

MACSQuant Analyzer 10, Miltenyi Biotec, Bergisch Gladbach, Germany

## Methods

### Preliminary Screening of Freezing Media

Three products/methodologies for freezing porcine PBMC were compared: Homemade freezing medium; PSC Cryopreservation kit, and CryoStor CS10. Fresh and frozen cells were assessed for viability and functionality by means of trypan blue staining and the IFN-γ ELISPOT (using PHA as a mitogenic stimulus) and IgG ELISPOT (using a R848+IL-2 cocktail).

Blood samples from eight commercial pigs of six weeks of age were collected in heparin tubes by jugular venipuncture. PBMC were isolated within 4h of collection by density-gradient centrifugation with Histopaque 1.077 in SepMate™ tubes. After two washes with PBS (400 x g, 10 min), PBMC of each animal were resuspended in SM1.

Cells collected in conical sterile tubes were stored overnight at 4°C. Afterwards, PBMC were counted using trypan blue staining and then were adjusted to the desired working concentrations. Half of the PBMC were used as fresh samples for ELISPOT assays, and the other half were frozen to repeat the same assays one month later.

For cryopreservation procedure with homemade freezing medium, PBMC were gently resuspended in 1mL of the freezing medium kept on ice and then transferred to cryovials. Cryovials were immediately distributed in controlled-grade freezing devices (−1°C/minute) and stored overnight at -80°C. The next day, samples were transferred to a liquid nitrogen tank at -196°C.

For PSC Cryopreservation kit and CryoStor CS10, PBMC were frozen following the manufacturer’s instructions. Thus, 1 mL of PSC Cryomedium (chilled at 4°C) was added dropwise to the PBMC, while gently rocking the tube back and forth, followed by gentle resuspension of the PBMC pellet. Then, samples were transferred to cryovials that were frozen as above. For CryoStor CS10, PBMC were softly resuspended in 1 mL (chilled at 4°C) and transferred to cryovials. Cryovials were distributed in controlled-grade freezing devices previously refrigerated at 4°C. Fifteen minutes after storing the cryovials at -80°C they were softly agitated. The next day, they were transferred to a liquid nitrogen tank at -196°C. In all cases, the concentration of PBMC was set at 20-25 x 10^6^ PBMC/cryovial.

One month later, all samples were thawed by immersion in a 37°C water bath. Then, PBMC were transferred to a conical sterile tube and diluted 1/10 in SM1. Samples were centrifuged (400 x g, 10 minutes), the supernatant was removed and PBMC were washed one more time under the same conditions. Finally, PBMC were resuspended again in SM1. For PSC Cryopreservation kit, samples were resuspended according to the manufacturer’s instructions; in this case, RevitaCell™ was added to the SM1 (100 μL in 10mL of medium). In all cases, cells were allowed to rest overnight at 37°C (5% CO_2_) with the cap loosened (14, 15). The next morning, PBMC were counted as above and adjusted to the desired working concentration.

The viability of fresh and frozen cells was assessed in a Neubauer chamber after trypan blue staining. Recovery rates were calculated for frozen cells as follows: (n° viable cells after thawing/n° viable cells before freezing) x 100. The functionality of fresh and frozen T and B-cells was assessed by means of the IFN-γ and IgG ELISPOT assays, respectively.

The IFN-γ ELISPOT was performed as previously described (16). Briefly, pre-wetted (200 μL/well PBS, 1 min) filter plates were coated with the P2G10 IFN-γ monoclonal antibody (diluted in carbonate–bicarbonate buffer at 1 μg/mL; 50 μL/well) and incubated overnight at 4°C. Plates were then washed with sterile PBS (200 μL/well) and blocked for 1h at 37°C with 100 μL/well of SM2. After removal of the blocking solution, PBMC were dispensed; 50,000 PBMC/well in 100 μL volume for PHA-stimulated wells (plus PHA 10 μg/ml in 100 μL SM1) or 500,000 PBMC/well in 100 μL volume plus 100 μL SM1 for negative control wells. After 20h of incubation (37°C; 5% CO_2_), PBMC were removed by washing plates five times using wash buffer (200 μL/well) allowing wells to soak well for 1 min in each wash step. Then, biotinylated detection antibody P2C11 was added at 0.5 μg/mL (50 μL/well diluted in the standard diluent) and incubated for 1h at 37°C. After that incubation, plates were washed as above and the reaction was revealed by incubation of plates with streptavidin-peroxidase diluted in standard diluent (final concentration 0.5 μg/mL; 50 μL/well; incubation 1h at 37°C), washing plates as above and addition of insoluble TMB (50 μL/well; 20 minutes, in the dark at room temperature). All tests were run in triplicates. A stereomicroscope binocular was used to read the spots. Frequencies of IFN-γ secreting cells (SC) were calculated by subtracting the counts of spots in unstimulated cells, from the counts in PHA-stimulated ones. Results were expressed as responding cells/10^6^ PBMC.

The IgG ELISPOT was carried out using a commercial kit. PBMC were split into two 1 mL aliquots in conical sterile polypropylene tubes. One of the aliquots was stimulated for 72 h (37°C, 5% CO2) with the polyclonal activator R848 and recombinant porcine IL-2 at 1 µg/mL and 10 ng/mL, respectively (16, 17). The other aliquot was kept as an unstimulated control. Aliquots were cultivated for three days prior to plating into ELISPOT. Then, cells were washed, resuspended in SM1, re-counted and adjusted to 100,000 cells/well. Plates previously pre-wetted (200 μL/well PBS, 1 min), coated with the MT421 monoclonal antibody and incubated overnight at 4°C, were washed and blocked. After removal of the blocking solution, PBMC were dispensed. Antibodies and streptavidin concentrations, as well as times of incubation, washing procedures, etc. were carried out following the manufacturer’s instructions. Captured IgG was visualized by the addition of the biotinylated antibody followed by the addition of streptavidin-peroxidase and insoluble TMB. All tests were run in triplicates. Frequencies of IgG-SC were calculated by subtracting the counts of spots in unstimulated cells, from the counts in stimulated ones. Results were expressed as responding cells/10^6^ PBMC. In all ELISPOT experiments, IgG and IFN-γ, all reagents were filtered (0.2 μm) before use.

### Evaluation of the Cryopreservation Impact on PBMC: Phenotyping and Responses to Mitogens and Specific Antigens

Five 4-week-old piglets (ear tags number 51, 53, 57, 59, 66) were immunized against PRRSV by vaccinating them with a modified live vaccine (PORCILIS PRRS^®^, 2 ml, intramuscular). Blood samples were collected in heparin tubes one month later. PBMC were obtained by gradient centrifugation as above; half of the PBMC were used as fresh samples for a set of analyses and the other half were frozen to repeat the same analyses after one month of frozen storage. Since results obtained during the preliminary screening pointed out that Cryostor CS10 provided the best results, it was selected as the freezing medium. The viability of fresh and frozen cells was assessed by trypan blue staining in a Neubauer chamber.

#### Phenotype of T and B-Cells

For phenotype characterization, fresh or frozen PBMC were initially incubated with supplemented PBS for 20 min on ice. Then, for T-cells labelling was carried out with antibodies against CD3, CD4α, and CD8α. Briefly, PBMC were incubated with the primary antibody anti-CD3 followed by an antibody anti-mouse IgG1-PE. In the next step, antibodies anti-CD4α:FITC and anti-CD8α:Alexa Fluor 647 were added. B-cells were labelled separately with an antibody anti-CD21conjugated to PE. Between each step, cells were washed twice with washing buffer and centrifuged at 500 x g for 5 min. All tests were run in triplicates. Samples were acquired on a MACSQuant Analyzer 10. Fluorescence minus one (FMO) controls, matched isotype controls, and background caused by secondary antibodies were used for gating and analysis. The acquired data were analysed using FCS Express 7.

#### Proliferation of T and B-Cells

For proliferation, fresh or frozen PBMC were labelled with CellTrace Violet at 5 μM according to the manufacturer’s instructions. Then, cells were suspended in SM1 at 2 × 10^6^ cells/ml with 100 μl/well plated in a 96-well U-bottom plate. Another 100 μl of SM1 or SM1 containing PRRSV isolate 3267 (MOI 0.5), PHA or ConA (10 μg/ml) were added as stimuli. After 5 days, cells were harvested and stained with anti-CD3, anti-CD4α, anti-CD8α and anti-CD21 antibody as described above. All tests were run in duplicates.

#### Frequencies of IFN-γ-Secreting Cells After Recall Antigen or Mitogen Stimulation Using ELISPOT

IFN-γ ELISPOT was done as described above, using both fresh and frozen PBMC. In this section, besides the PHA response, the antigen-specific (PRRSV) IFN-γ-SC frequencies were also measured. For this purpose, 500,000 cells/well were stimulated with the PRRSV strain 3267 diluted in SM1 at a multiplicity of infection (MOI) of 0.1. PHA-stimulated cultures and PBMC incubated in SM1 were included as positive and negative controls, respectively as described above. Frequencies of PRRSV-specific and PHA-stimulated IFN-γ-SC were calculated by subtracting the counts of spots in unstimulated cells from the counts in stimulated ones. Results were expressed as responding cells/10^6^ PBMC.

#### IgG ELISPOT

IgG ELISPOT to measure polyclonal responses were performed as described above.

### Statistical Analysis

Statistics were performed using StatsDirect v2.7.7. Mann-Whitney U- and Wilcoxon’s signed ranks, or Kruskal-Wallis (Dwass–Steel–Chritchlow–Fligner method for multiple comparisons) non-parametric tests were used for comparisons of means between two or more sets of data, respectively.

## Results

### Preliminary Product Screening to Freeze Porcine PBMC

Using PBMC from eight pigs, the average viabilities for homemade freezing medium, PSC Cryopreservation kit, and CryoStor CS10 after thawing were 96 ± 2.1%^a^, 89 ± 3.2%^b^, and 97 ± 1.1%^a^, respectively (*p*<0.05). Regarding recovery rates, no significant differences were observed (**Table 1**).

**Table 1:** Viability and recovery rate (percentages) for the preliminary screening of the three freezing media assayed (n=8).

	Homemade freezing media	PSC Cryopreservation kit	CryoStor CS10
**Viability (%)**	96.0 ± 2.1^a^	89.0 ± 3.2^b^	97.0 ± 1.1^a^
	(93.2 - 99.0)	(85.0 - 95.1)	(95.2 - 98.3)
**Recovery rate (%)**	70.0 ± 5.5	70.5 ± 4.7	71.1 ± 5.1
	(61.1 - 76.9)	(62.5 - 76.3)	(61.8 - 77.3)

^a, b^Superscript letters show significant differences (p<0.05).

Means ± standard deviations (SD) and ranges.

For fresh cells, the average frequency of IFN-γ-SC stimulated by PHA was 706.0 ± 86.7 per 10^6^ PBMC. After freezing, although the average value dropped to 674.6 ± 64.9 for homemade freezing medium (variance percentage compared to fresh PBMC was -4.0%: ranging from -15.4% to +6.5% in individual samples), 634.6 ± 53.7 for the PSC Cryopreservation kit (-9.7%, ranging from -15.7% to -1.7%; p=0.07), and 670.1 ± 77.0 for CryoStor CS10 (-5.0%, ranging from -8.1% to +1.5%), the changes were not significant. None of the cryopreservation procedures increased the background IFN-γ production (**Supplementary Table 1**).

In the IgG ELISPOT, the average frequency of IgG secreting cells for fresh PBMC stimulated with the mitogenic cocktail (R848 + IL-2) was 490.6 ± 186.4 per 10^6^ PBMC. After freezing, the average values were 408.0 ± 158.9 for homemade freezing medium (variance percentage compared to fresh PBMC was -16.9%, ranging from -21% to -11.8% in individual samples), 386.4 ± 145.0 for the PSC Cryopreservation kit (-21.0%, ranging from -25.7% to -14.4%), and 419.2 ± 161.0 for CryoStor CS10 (-14.8%, ranging from -21.8% to -9.4%). Although means of variance percentage were lower compared to those observed for IFN-γ, significant differences between fresh and frozen PBMC for IgG were not found (**Supplementary Table 1**).

Overall, the freezing process had a small impact on cell viability after using any of the three products. The impact on T cell functionality -measured as the response against PHA- was almost negligible when using homemade freezing medium or CryoStor CS10. For B-cells, no significant differences were observed between fresh and frozen PBMC for any of the freezing products evaluated. Eventually, Cryostor CS10 was used to further examine the cryopreservation impact on T and B phenotypes and antigen-specific responses.

### Evaluation of the Impact of Cryopreservation on PBMC

#### Impact of Cryopreservation on Viability and Recovery Rates

In the second study, PBMC viability, as assessed by trypan blue staining, was 99.2 ± 0.8 in fresh samples, while the average values of frozen PBMC replicas were 94%, 92%, 93%, 97%, and 93%. No replicas of any animal dropped below 88%. Mean of recovery rates was 72.3%.

#### Impact of Cryopreservation on the Phenotype and Proliferation of T-Cells

**Figure 1:**
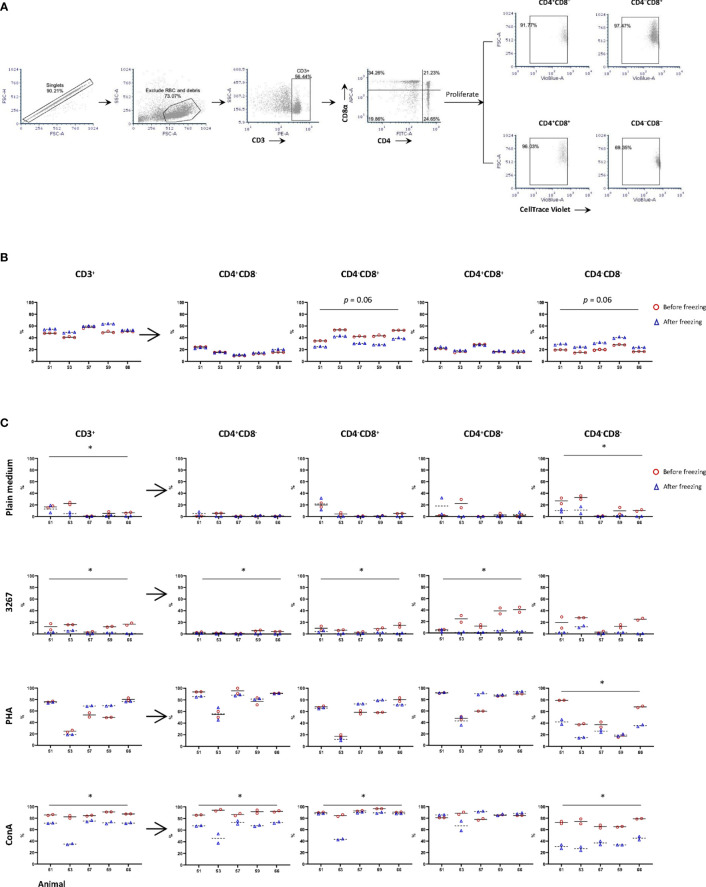
Impact of cryopreservation on phenotype and proliferation of T lymphocytes. **(A)** Gating hierarchy of T cells. T cells were gated as CD3^+^ cells, then further divided into CD4^+^CD8α^–^, CD4^–^CD8α^+^, CD4^+^CD8α^+^, and CD4^–^CD8α^–^. The proliferation of CD3^+^ cells and the four subsets were assessed by the proportion of CellTrace Violet^low^ cells within each population; **(B)** Proportion of CD3^+^ cells and different subsets of T cells in fresh (red circles) and frozen (blue triangles) PBMC. Five animals were examined with three replicas per animal, and the means were indicated as solid and dashed lines, respectively; **(C)** Proliferation of CD3^+^ cells and subsets CD4^+^CD8α^–^, CD4^–^ CD8α^+^, CD4^+^CD8α^+^, and CD4^–^ CD8α^–^ using fresh (red circles) and frozen (blue triangles) PBMC. PBMC were labeled by CellTrace Violet and then stimulated by SM1 (plain medium), PRRSV strain 3267 (3267), Phytohemagglutinin (PHA), or Concanavalin A (Con A) for five days before being stained for CD3/CD4/CD8. Two replicas were shown for both fresh and frozen PBMC with the means indicated as solid and dashed lines, respectively. Statistical significance was calculated by the Wilcoxon’s signed ranks test with the average value of each animal, *p < 0.05.

Phenotypically, TCR-αβ T-cells (CD3^+^) were roughly subdivided into naïve (CD4^+^CD8^–^), cytotoxic (CD4^–^CD8^+^), memory (CD4^+^CD8^+^) T-cells, and a CD4/CD8 double negative subset that have not been well defined (18) (gating hierarchy as shown in **Figure 1A**). In fresh PBMC, CD3^+^ cells (T-lymphocytes) accounted on average 49.3 ± 6.1% (after excluding debris and red blood cells), compared to 56.0 ± 5.7% in frozen cells. Within CD3^+^ cells, freezing resulted in an equivalent proportion of CD4^+^CD8^–^ and CD4^+^CD8^+^ subsets, but an increase (p = 0.06) of CD4^–^CD8^–^ subset and, accordingly, a decrease (*p* = 0.06) in CD4^–^CD8^+^ subset compared to the counterparts in freshly isolated cells (**Figure 1B**).

When PBMC were stimulated with PRRSV 3267 (MOI 0.5) for five days, it was shown that the proliferation of CD3^+^ cells (gating hierarchy as shown in **Figure 1A**) was impaired by freezing, decreasing from 12.2 ± 5.4% (fresh cells) to 2.2 ± 2.0% (frozen cells) (p < 0.05) (**Figure 1C**). The impairment was also marked (*p* < 0.05) for subsets defined by CD4/CD8 within CD3^+^ cells (**Figure 1C**). Thus, freezing caused a clear reduction in the proliferation of CD4 and CD8 single-positive cells, as well as CD4/CD8 double-negative cells (*p* < 0.05) after stimulation by Con A. In contrast, when cells were stimulated with PHA, the reduction of the proliferation was only observed in CD4/CD8 double-negative cells (**Figure 1C**).

To assess whether an impairment of CD172a+ cells due to cryopreservation could explain the lower frozen PBMC proliferation, cells were stained for CD172a/CD3/CD21/live-dead Near-IR. The examination of CD172a+ cells showed that their viability after the overnight resting differed by less than 2% from that obtained for CD3+ T-cells and the whole PBMC.

#### Impact of Cryopreservation on IFN-γ ELISPOT: Responses to Recall Antigen and Mitogens

**Figure 2:**
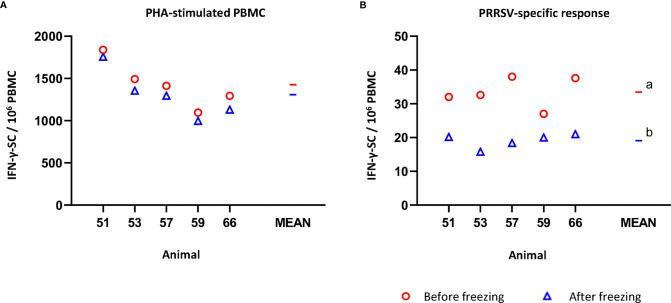
Impact of cryopreservation on frequencies of IFN-γ-secreting cells (SC) by means of ELISPOT. **(A)** PHA-stimulated IFN-γ-SC by million PBMC. **(B)** PRRSV-specific IFN-γ-SC by million PBMC. Results obtained for each pig using fresh PBMC are shown as red circles (mean is indicated by a red line), while results obtained with frozen cells are shown as blue triangles (mean is indicated by a blue line). Statistical significance was calculated by the Mann-Whitney U-test.^a,b^ Superscript letters show significant differences (p < 0.05).

The average number of IFN-γ-SC in PHA-stimulated fresh PBMC was 1426.6 ± 274.9 per 10^6^ PBMC (**Figure 2A**). After freezing, the average value was 1307.7 ± 288.5 (non-significant). Individually, the reduction in the number of IFN-γ-SC in PHA-stimulated never exceeded -13% (mean of variance percentage = -8.6, ranging from -4.3% to -12.8%). However, when the response to recall antigen was measured, the average frequency of PRRSV-specific IFN-γ-SC was significantly lower in frozen than in fresh cells (19.1 ± 2.4 versus 33.4 ± 4.4; p<0.05) (**Figure 2B**). On average, a 41.5% reduction was observed in frozen cells; individually, from -21.1% to -54.0% (**Supplementary Table 2**).

#### Impact of Cryopreservation on Phenotype and Proliferation of B-Cells

**Figure 3:**
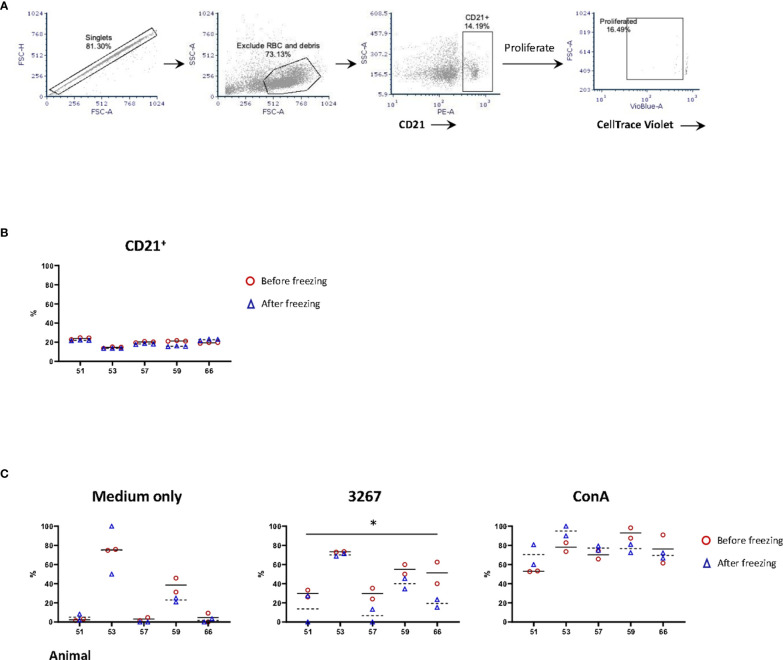
Impact of cryopreservation on phenotype and proliferation of B lymphocytes. **(A)** Gating hierarchy of B cells. B cells were gated as CD21^+^ cells. The proliferation was assessed by the proportion of CD21^+^ cells with CellTrace Violet^low^; **(B)** Comparison of the proportion of CD21^+^ cells in fresh (red circles) and frozen (blue triangles) PBMC. Five animals in three replicas were shown. The means were indicated with solid (fresh PBMC) and dashed lines (frozen PBMC), respectively; **(C)** Proliferation of CD21^+^ cells. PBMC (labeled with CellTrace Violet) were stained for CD21 after five days of stimulation by SM1 (plain medium), PRRSV strain 3267 (3267), or Concanavalin A (Con A). Two replicas were shown for both fresh (red circles) and frozen (blue triangles) PBMC with the means indicated as solid and dashed lines, respectively. Statistical significance was calculated by the Wilcoxon’s signed ranks test with the average value of each animal, *p < 0.05.

The proportions of B-cells (gating hierarchy as shown in **Figure 3A**) before or after freezing were similar (14.5% to 24.0% before freezing versus 13.7% to 22.7% after freezing, non-significant) (**Figure 3B**). The proliferation of B-cells (gating hierarchy as shown in **Figure 3A**) was impaired in frozen cells stimulated with the recall antigen PRRSV (p < 0.05), but not in Con A-stimulated cultures. (**Figure 3C**). Of note, the proportion of CD21^+^ cells in the 3267- and ConA-stimulated cultures was very low, < 2% and < 0.6%, respectively. Also, there was an apparent discrepancy between animals.

#### Impact of Cryopreservation on IgG ELISPOT: Responses to Mitogens

**Figure 4:**
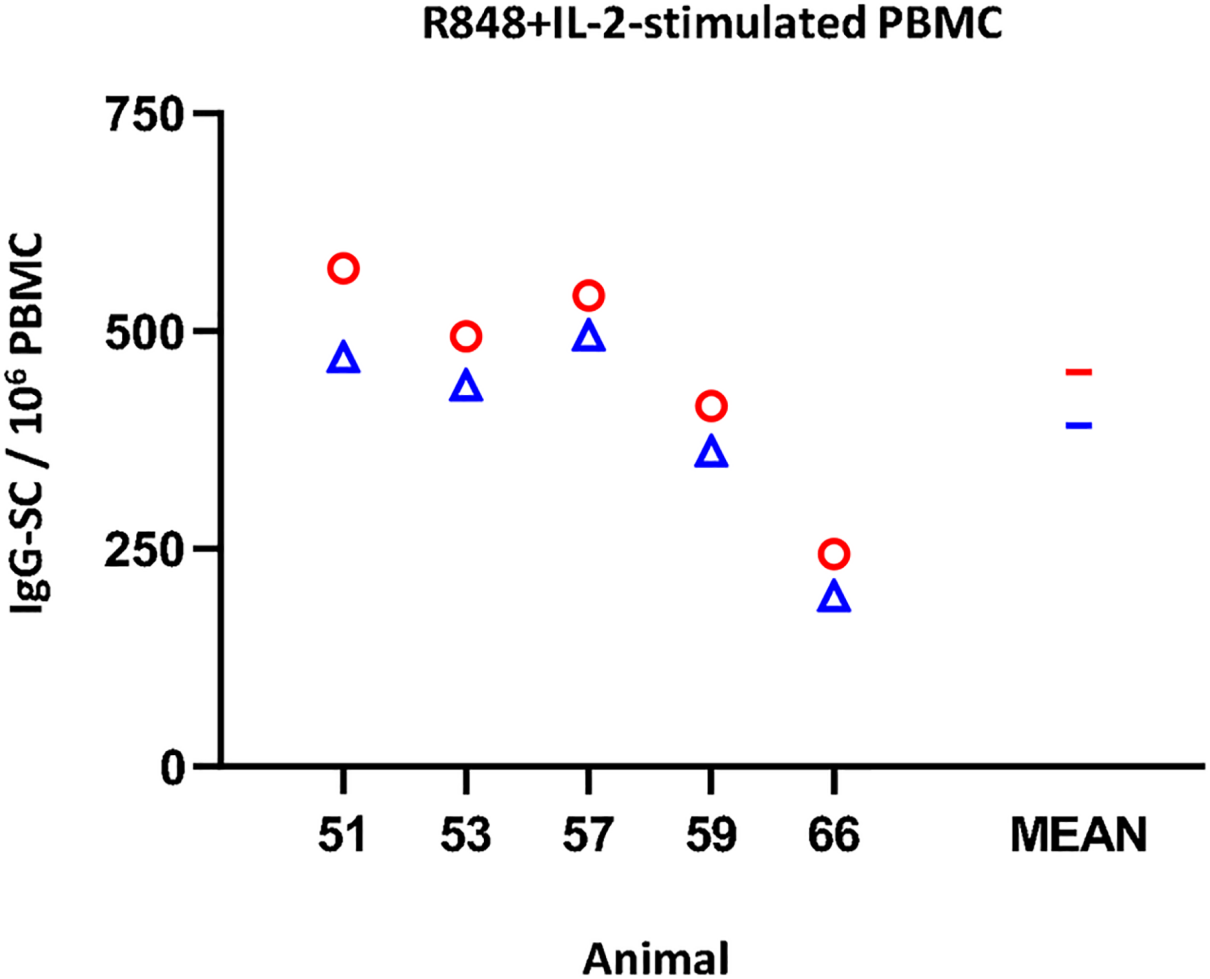
Impact of cryopreservation on frequencies of IgG-secreting cells (SC) by means of ELISPOT. R848+IL-2-stimulated IgG-SC by million PBMC. Results obtained for each pig using fresh PBMC are shown as red circles (mean is indicated by a red line), while results obtained with frozen cells are shown as blue triangles (mean is indicated by a blue line).

Regarding the IgG ELISPOT (**Figure 4**), the response to the mitogenic cocktail (R848 + IL-2) resulted in 453 ± 131.1 IgG-SC per 10^6^ freshly isolated PBMC, comparable to the average value of frozen cells, 392.2 ± 120.5 (non-significant). Variance percentage for the five examined individuals ranged from -8.5% to -19.7% (mean = -14.0).

## Discussion

The use of frozen PBMC in pig immunology is a common procedure. Most often this is a need arising from the complex logistics required for processing samples collected in a farm located far from the analysis laboratory. However, there is very little information regarding the performance of frozen porcine PBMC compared to freshly isolated ones.

In the present study, the first step was to compare several cryopreservation media. The results of our comparison indicated that the viability after recovery was similar for all three specific freezing media (≥89% on average) (**Table 1**), although cells frozen with PSC Cryopreservation kit showed a trend to perform worse in the IFN-γ ELISPOT (**Supplementary Table 1**). It has been reported that the viability of PBMC below 70-80% seriously impaired the results of functional tests (19–21). Regarding recovery rates, no obvious impact was observed for any of the cryopreservation procedures (**Table 1**), obtaining similar results to those described by Liang and collaborators (2019) (22), who found a mean of 73.7% when comparing five methods. With our results, homemade freezing medium, PSC cryopreservation kit, and CryoStor CS10 fulfilled requirements of viability and recovery. In the following experiments, CryoStor CS10 was used for the sake of convenience.

The additional analyses using a recall antigen or different mitogens showed that the impact of freezing was more evident when the recall responses were examined, as indicated by the reduction of proliferation and the number of spots produced in IFN-γ ELISPOT (**Figures 1C** and **2B**, respectively). Since the viability of CD172^+^ cells, which comprised the majority of antigen presenting cells, did not differ from that of CD3^+^ cells or the whole PBMC, the poorer recall response was supposed not caused by extra extent damage of CD172^+^ cells. Such similar studies using human or mice PBMC are still controversial (3–10, 23). Ford et al. (10) reported a decrease in the frequency of antigen-specific IFN-γ producing CD4^+^ T-cells in malaria vaccine studies. According to the authors, the impairment affected mostly short-term IFNγ-producing effector memory CD4^+^ T-cells, while the potentially central memory cells seemed to be retained in cryopreserved PBMC. This could be consistent with our observations using porcine cells. Firstly, animals used in the present study were vaccinated against PRRSV one month before sampling, so it would be expectable to have recently developed memory cells (not long-lived yet). Secondly, the effector memory T-cells are thought to be the main source of IFN-γ among human CD4^+^ memory T-cells (revised by 24). Some preliminary evidence was also observed *via in vitro* re-stimulation of PBMC from PRRSV-infected pigs (25).

A pre-stimulation step before performing the assay has been suggested to improve the sensitivity of the recall responses in the IFN-γ ELISPOT, particularly using frozen cells (26). Interestingly, in a subsequent experiment in which we compared the performance of frozen PBMC from PRRSV-vaccinated pigs using pre-stimulation and no-pre-stimulation, we observed a significant increase in the frequency of PRRSV-specific IFN-γ-SC from two animals, while no improvement was observed from the rest (data not shown). Therefore, as in Smith et al. (26), the improvement observed performing the pre-stimulation step depended on the individual.

It is worth noting that, in our case, the proliferative response of double positive CD4/CD8α T-cells to the recall antigen was largely impaired when PBMC were frozen (**Figure 1C**). Porcine CD4/CD8 double positive cells account for a large proportion of memory cells (27), which have been demonstrated as the primary source of IFN-γ when responded to the PRRSV recall stimulation (28). The expression of CD27 further divides them into central (TCM, CD4+/CD8α+/CD27+) and effector memory T-cells (TEM, CD4^+^/CD8α^+^/CD27^–^) (27). As reported by Kick et al. (25), the response of memory cells might be associated with the clearance of PRRSV viremia when pigs were challenged. Upon *in vitro* re-stimulation, TCM proliferated at a higher level, while TEM are more potent in producing IFN-γ and TNF-α. A specific impairment of the function of these memory cells after freezing may undermine their role in response to different antigens, at least to PRRSV. But other studies on human PBMC indicated that freezing did not have a substantial effect on the recall responses (4–6). Further confirmation would require a specific analysis of the naïve, TCM and TEM compartments using animals at different times of immunisation (short- and long-term after antigen exposure).

When using mitogens for T-cells or polyclonal activation of B-cells, the picture was different (**Figures 2A**, **4**, respectively; **Supplementary Table 2**). The impact of freezing seemed to be of minor importance, if any, for polyclonally activated PBMC used in the IFN-γ of IgG ELISPOT. Li et al. (12) suggested that freezing of porcine PBMC could even result in a slight increase in the cytokine production upon PMA stimulation.

In our case, when PHA was used, the proliferation of T-cells was slightly affected. But the individual variation was noticeable, and in some cases, cells that were frozen showed even a higher proliferation (**Figure 1C**). In contrast, Koch et al. (11) reported a decreased proliferation, as measured by 3H-thymidine incorporation, but, in Koch’s study, PBMC were not rested after thawing. In others papers it has been shown that resting PBMC after thawing improved the proportion of T-cells, particularly CD4 (15).

In striking contrast, proliferation after stimulation with Con A was significantly impaired in cells that were frozen (**Figure 1C**). The precise mechanisms by which PHA and Con A induce T-cell proliferation are poorly understood. Besides being lectins and acting by crosslinking, activation of T-cells by Con A and PHA might happen *via* distinct molecular machinery. For example, CD28 was assumed to be a co-stimulatory signal during Con A stimulation (29), which has not been proved for PHA. The precise reason by which freezing affected cultures activated with one or the other lectin cannot be ascertained in the present study.

The last effect related to the freezing was a decrease in the proportion of CD21^+^ cells. Reimann et al. (3) determined that freezing resulted in a decrease of the proportion of CD19^+^ cells in PBMC from HIV patients. The causes of such loss were not resolved at that moment and are also unable to be determined through our results. But, in any case, the loss of CD21^+^ cells did not significantly affect the IgG ELISPOT.

To conclude, our results suggest that frozen porcine PBMC are mostly suitable for immunophenotyping and functional testing as long as mitogens are used. For recall antigen stimulation, freezing had a significant impact on the magnitude of the response, although responding animals could be identified. Nevertheless, same PBMC condition (fresh or frozen) should be used within a given study/trial to ensure comparability of the results. Given that freezing is a common treatment when working with porcine PBMC, it would be advisable to further characterize the response of naïve and memory cells at different times of the immune response.

## Data Availability Statement

The raw data supporting the conclusions of this article will be made available by the authors, without undue reservation.

## Ethics Statement 

The animal study was reviewed and approved by Comissió d’ètica en experimentació animal i humana (2501CEEA-UAB).

## Author Contributions

YL contributed to the design and the execution of part of the experiments, the interpretation of the data, and the writing of the manuscript. EM contributed to the design of the experiments, the interpretation of the data, and the writing of the manuscript. ID contributed to the design and the execution of part of the experiments, the interpretation of the data, and the writing of the manuscript. All authors contributed to the article and approved the submitted version.

## Funding

This work was partially supported by the National Institute of Research and Agricultural and Food Technology (INIA, reference E-RTA2015-0003-C02-01) and by funds of the Laboratori Veterinari de Diagnosi de Malalties Animals of the Universitat Autònoma de Barcelona (UAB).

## Conflict of Interest

The authors declare that the research was conducted in the absence of any commercial or financial relationships that could be construed as a potential conflict of interest.

## Publisher’s Note

All claims expressed in this article are solely those of the authors and do not necessarily represent those of their affiliated organizations, or those of the publisher, the editors and the reviewers. Any product that may be evaluated in this article, or claim that may be made by its manufacturer, is not guaranteed or endorsed by the publisher.
