# Supplementary material for: Impact of Cryopreservation on Viability, Phenotype, and Functionality of Porcine PBMC
Source: Front Immunol. 2021 Nov 29;12:765667. doi: 10.3389/fimmu.2021.765667 (PMC8666977; doi:10.3389/fimmu.2021.765667)
Supplement: Supplementary file 1 [file Table_1.docx]

**Supplementary table 1. Raw data for** **IFN-γ and IgG ELISPOT from Experiment 1: “Preliminary screening of freezing media”.** Results (mean of three replicas by stimulus) are shown as IFN-γ- or IgG-secreting cells / 10^6^ PBMC.

**IFN-γ ELISPOT**

|  | **Fresh PBMC** | | | **Homemade freezing media** | | | | **PSC Cryopreservation kit** | | | | **CryoStor CS10** | | | |
| --- | --- | --- | --- | --- | --- | --- | --- | --- | --- | --- | --- | --- | --- | --- | --- |
| **Animal** | **None^1^** | **PHA^2^** | **PHA**  **Res^3^** | **None** | **PHA** | **PHA**  **Res** | **Var %**  **PHA^4^** | **None** | **PHA** | **PHA**  **Res** | **Var %**  **PHA** | **None** | **PHA** | **PHA**  **Res** | **Var %**  **PHA** |
| **1** | 4 | 864 | **860** | 5 | 770 | **765** | **-11** | 4 | 729 | **725** | **-15.7** | 8 | 808 | **800** | **-7** |
| **2** | 5 | 625 | **620** | 6 | 656 | **650** | **+4.8** | 8 | 608 | **600** | **-3.3** | 3 | 573 | **570** | **-8.1** |
| **3** | 2 | 702 | **700** | 2 | 707 | **705** | **+0.7** | 0 | 620 | **620** | **-11.5** | 2 | 652 | **650** | **-7.2** |
| **4** | 3 | 613 | **610** | 0 | 650 | **650** | **+6.5** | 2 | 602 | **600** | **-1.7** | 0 | 585 | **585** | **-4.1** |
| **5** | 8 | 658 | **650** | 4 | 554 | **550** | **-15.4** | 5 | 590 | **585** | **-10** | 3 | 663 | **660** | **+1.5** |
| **6** | 6 | 694 | **688** | 4 | 656 | **652** | **-5.2** | 2 | 597 | **595** | **-13.5** | 2 | 662 | **660** | **-4.1** |
| **7** | 2 | 722 | **720** | 3 | 703 | **700** | **-2.8** | 2 | 647 | **645** | **-10.4** | 2 | 687 | **685** | **-4.9** |
| **8** | 3 | 803 | **800** | 4 | 729 | **725** | **-9.4** | 3 | 710 | **707** | **-11.6** | 6 | 757 | **751** | **-6.2** |
| **MEAN** | 4.1 | 710.1 | **706.0** | 3.5 | 678.1 | **674.6** | **-4.0** | 3.2 | 637.8 | **634.6** | **-9.7** | 3.2 | 673.3 | **670.1** | **-5.0** |
| **SD^5^** | 2.1 | 86.3 | **86.7** | 1.8 | 65.1 | **64.9** |  | 2.4 | 53.5 | **53.7** |  | 2.5 | 79.1 | **77.0** |  |

**IgG ELISPOT**

|  | **Fresh PBMC** | | | **Homemade freezing media** | | | | **PSC Cryopreservation kit** | | | | **CryoStor CS10** | | | |
| --- | --- | --- | --- | --- | --- | --- | --- | --- | --- | --- | --- | --- | --- | --- | --- |
| **Animal** | **None** | **IL-2**  **R848^6^** | **Res^7^** | **None** | **IL-2**  **R848** | **Res** | **Var %^8^** | **None** | **IL-2**  **R848** | **Res** | **Var %** | **None** | **IL-2**  **R848** | **Res** | **Var %** |
| **1** | 5 | 680 | **675** | 12 | 573 | **561** | **-16.9** | 9 | 529 | **520** | **-23** | 16 | 604 | **588** | **-12.9** |
| **2** | 55 | 555 | **500** | 41 | 456 | **415** | **-17** | 43 | 432 | **389** | **-22.2** | 33 | 486 | **453** | **-9.4** |
| **3** | 15 | 795 | **780** | 7 | 677 | **670** | **-14.1** | 12 | 614 | **602** | **-22.8** | 17 | 677 | **660** | **-15.4** |
| **4** | 15 | 285 | **270** | 17 | 242 | **225** | **-16.7** | 22 | 253 | **231** | **-14.4** | 11 | 222 | **211** | **-21.8** |
| **5** | 0 | 300 | **300** | 5 | 247 | **242** | **-19.3** | 0 | 223 | **223** | **-25.7** | 1 | 266 | **265** | **-11.7** |
| **6** | 60 | 680 | **620** | 40 | 543 | **503** | **-18.9** | 48 | 566 | **518** | **-16.5** | 41 | 556 | **515** | **-16.9** |
| **7** | 30 | 378 | **348** | 37 | 344 | **307** | **-11.8** | 31 | 312 | **281** | **-19.3** | 28 | 327 | **299** | **-14.1** |
| **8** | 20 | 452 | **432** | 25 | 366 | **341** | **-21** | 22 | 349 | **327** | **-24.3** | 23 | 386 | **363** | **-16** |
| **MEAN** | 25.0 | 515.6 | **490.6** | 23.0 | 431.0 | **408.0** | **-16.9** | 23.4 | 409.7 | **386.4** | **-21.0** | 21.2 | 440.5 | **419.2** | **-14.8** |
| **SD** | 22.0 | 191.3 | **186.4** | 14.9 | 158.2 | **158.9** |  | 16.6 | 148.1 | **145.0** |  | 12.7 | 165.7 | **161.0** |  |

^1^ Unstimulated. ^2^ PHA-stimulation. ^3^ PHA result = PHA-stimulation – Unstimulated. ^4^ Variance percentage PHA = [(PHA result from fresh cells – PHA result from frozen cells) / PHA result from fresh cells] *100. ^5^ Standard deviation. ^6^ IL-2+R848-stimulation. ^7^ IL-2+R848 result = IL-2+R848-stimulation – Unstimulated. ^8^ Variance percentage IL-2+R848 = [(IL-2+R848 result from fresh cells - IL-2+R848 result from frozen cells) / IL-2+R848 result from fresh cells] *100.

**Supplementary table 2. Raw data for IFN-γ and IgG ELISPOT from Experiment 2: “Evaluation of the cryopreservation impact on PBMC: phenotyping and responses to mitogens and specific antigens”.** Results (mean of three replicas by stimulus) are shown as IFN-γ- or IgG-secreting cells / 10^6^ PBMC.

**IFN-γ ELISPOT**

|  | **Fresh PBMC** | | | | | **Frozen PBMC** | | | | | | |
| --- | --- | --- | --- | --- | --- | --- | --- | --- | --- | --- | --- | --- |
| **Animal** | **None^1^** | **PHA^2^** | **PHA**  **Res^3^** | **PRRSV^4^** | **PRRSV**  **Res^5^** | **None** | **PHA** | **PHA**  **Res** | **Var %**  **PHA^6^** | **PRRSV** | **PRRSV**  **Res** | **Var %**  **PRRSV^7^** |
| **51** | 15 | 1854 | **1839** | 47 | **32** | 12 | 1771 | **1759** | **-4.3** | **32.3** | **20.5** | **-36.6** |
| **53** | 7 | 1500 | **1493** | 40 | **33** | 4.5 | 1360 | **1355.5** | **-9.2** | **19.7** | **15.2** | **-54** |
| **57** | 2 | 1414 | **1412** | 40 | **38** | 3 | 1299 | **1296** | **-8.2** | **22** | **19** | **-50** |
| **59** | 5 | 1100 | **1095** | 32 | **27** | 1 | 1001.3 | **1000.3** | **-8.6** | **22.3** | **21.3** | **-21.1** |
| **66** | 1 | 1295 | **1294** | 38 | **37** | 1 | 1128.7 | **1127.7** | **-12.8** | **21** | **20** | **-46** |
| **MEAN** | 6.0 | 1432.6 | **1426.6** | 39.4 | **33.4** | 4.3 | 1312 | **1307.7** | **-8.6** | **23.4** | **19.1** | **41.5** |
| **SD^8^** | 5.7 | 279.3 | **274.9** | 5.4 | **4.4** | 4.5 | 292.9 | **288.5** |  | **5.0** | **2.4** |  |

**IgG ELISPOT**

|  | **Fresh PBMC** | | | **Frozen PBMC** | | | |
| --- | --- | --- | --- | --- | --- | --- | --- |
| **Animal** | **None** | **IL-2**  **R848^9^** | **Res^10^** | **None** | **IL-2**  **R848** | **Res** | **Var %^11^** |
| **51** | 12 | 584 | **572** | 14 | 484 | **470** | **-17.8** |
| **53** | 6 | 500 | **494** | 2 | 440 | **438** | **-11.3** |
| **57** | 9 | 550 | **541** | 7 | 502 | **495** | **-8.5** |
| **59** | 26 | 440 | **414** | 22 | 384 | **362** | **-12.6** |
| **66** | 6 | 250 | **244** | 8 | 204 | **196** | **-19.7** |
| **MEAN** | 11.8 | 464.8 | **453** | 10.6 | 402.8 | **392.2** | **-14.0** |
| **SD** | 8.3 | 131.8 | **131.1** | 7.7 | 120.1 | **120.5** |  |

^1^ Unstimulated. ^2^ PHA-stimulation. ^3^ PHA result = PHA-stimulation – Unstimulated. ^4^ PRRSV-stimulation. ^5^ PRRSV result = PRRSV-stimulation – Unstimulated ^6^ Variance percentage PHA = [(PHA result from fresh cells – PHA result from frozen cells) / PHA result from fresh cells] *100. ^7^ Variance percentage PRRSV = [(PRRSV result from fresh cells - PRRSV result from frozen cells) / PRRSV result from fresh cells] *100. ^8^ Standard deviation.^9^ IL-2+R848-stimulation. ^10^ IL-2+R848 result = IL-2+R848-stimulation – Unstimulated. ^11^ Variance percentage IL-2+R848 = [(IL-2+R848 result from fresh cells - IL-2+R848 result from frozen cells) / IL-2+R848 result from fresh cells] *100.
